# Supplementation of Arginine or N-Carbamylglutamate Affects Jejunum Development, Global Arginine Bioavailability Ratio, and Stress-Related Indices in Young Rex Rabbits

**DOI:** 10.3390/ani15101354

**Published:** 2025-05-08

**Authors:** Feng Qin, Linlin Zhang, Le Shao, Jian Li, Jie Yang, Pin Zhai, Xia Zhang

**Affiliations:** 1 Institute of Animal Science, Jiangsu Academy of Agricultural Sciences, Nanjing 210014, China; fqin1983@163.com (F.Q.); dshaole1981@163.com (L.S.); skygohealth@hotmail.com (J.L.); 19980022@jaas.ac.cn (J.Y.); lotus_96@163.com (X.Z.); 2 Key Laboratory of Crop and Livestock Integrated Farming, Ministry of Agriculture and Rural Affairs, Nanjing 210014, China; 3 College of Life Science, Longyan University, Longyan 364012, China; zhanglinlin0902@163.com

**Keywords:** young rex rabbits, arginine, N-carbamylglutamate, nitric oxide, global arginine bioavailability ratio

## Abstract

This study aimed to investigate the effects of arginine (Arg) or N-carbamylglutamate (NCG) on jejunum development, the global arginine bioavailability ratio (GABR), and stress-related indices in young rex rabbits. Forty-five litters of newborn rabbits with similar litter weights and sizes were randomly divided into five groups and fed a basal diet (con group), basal diet + 0.3% Arg (0.3% Arg group), basal diet + 0.6% Arg (0.6% Arg group), basal diet + 0.03% NCG (0.03% NCG group), or basal diet + 0.06% NCG (0.06% NCG group). After weaning at 35 days, eight healthy young rabbits with similar body weights were selected from each group and slaughtered on the 36th day. Serum and jejunum samples were collected for index analysis. Arg or NCG significantly affected the jejunum structure development in the young rabbits. The villus height (V) in the treatment groups was significantly higher than that in the con group (*p* < 0.05) and was highly significantly improved in the 0.6% Arg group (*p* < 0.01). The crypt depth (C) in 0.6% Arg and 0.06% NCG groups was significantly lower than that in the con, 0.3% Arg, and 0.03% NCG groups (*p* < 0.05). Compared with the con group, the V/C ratio was significantly increased in the treatment groups (*p* < 0.05) and was excessively and significantly increased in the 0.6% Arg and 0.06% NCG groups (*p* < 0.01). Compared with the con group, the Nitric-Oxide (NO) levels and inducible Nitric-Oxide Synthase (iNOS) activity in serum were significantly increased in the treatment groups. The NO levels in the jejunum were also increased in the treatment groups; however, there were no significant differences (*p* > 0.05). The iNOS activity and mRNA expression in the jejunum of the 0.6% Arg and 0.06% NCG groups were significantly higher than those in the con group (*p* < 0.05). Compared with the con group, the concentration of serum corticosterone in the 0.3% Arg, 0.6% Arg, and 0.06% NCG groups was significantly reduced. Adding Arg or NCG to the basal diet significantly increased the concentration and gene *mRNA* expression levels of heat shock protein 70 (HSP70) in the jejunum (*p* < 0.05). The expression level in the 0.6% Arg and 0.06% NCG groups was significantly higher than that in the con group (*p* < 0.01). At 36 days, Arg and NCG improved the GABR. In the 0.6% Arg group, the GABR was increased by 16.92%. The GABR was <0.8 at the time of weaning. The Arg levels in the body did not meet the needs of the young animals. In the trial, Arg or NCG supplementation in the diet significantly increased iNOS activity and gene *mRNA* expression, promoting NO synthesis. Thus, it can improve jejunal morphological and structural development. Adding Arg or NCG increased HSP70 *mRNA* expression levels, enhanced intestinal stress tolerance, and improved intestinal health. During the lactation period, adding Arg or NCG increased the GABR, but the GABR was <0.8 during weaning.

## Simple Summary

Rabbits are important economic and experimental animals. At present, the main challenge facing rabbit production is maintaining gut health during the post-weaning period. This study focused on the gut health of weaning rabbits by adding arginine (Arg) or N-carbamylglutamate (NCG) to their diet. The assessment was performed from jejunum development, focusing on stress-related indices. Adding 0.6% Arg or 0.06% NCG can improve jejunum morphological and structural development. They also up-regulated HSP70 mRNA expression, enhanced intestinal stress tolerance, and improved gut health. During the lactation period, adding 0.6% Arg or 0.06% NCG increased the GABR, but GABR was <0.8 during weaning, which could be crucial for predicting diseases and also reflect the effect of arginine or NO on the body.

## 1. Introduction

Currently, the rabbit industry is facing a critical period. The limitation of the prophylactic use of antibiotics in animal production in recent years has led to an increase in rabbit mortality in many farms, often making production almost economically unsustainable. In this respect, the main challenge in rabbit production is maintaining gut health during the post-weaning period [1,2,3]. In this period, the digestive tract is very vulnerable and susceptible to the proliferation of bacterial pathogens and parasitic protozoa. How to improve gut health is the focus of nutritionists. Dietary strategies are particularly key in the suckling and post-weaning period.

Arginine (Arg) is an essential amino acid for young animals and is crucial to nutrition, physiology, and immunity. Adding arginine to the diet can reportedly improve intestinal development and function, promote intestinal health, alleviate stress, and further increase growth performance [4,5,6]. However, the price of arginine is too high. This raises the question, are there more economical alternatives? N-carbamylglutamate (NCG) is an analog of N-acetylglutamate, which can effectively promote the synthesis of endogenous arginine, and enhance the function of arginine. Nitric oxide (NO) may play a vital role in the synthesis of arginine to improve intestinal development and relieve stress. Gookin et al. [7] reported that arginine-generated NO had a reparative effect on severely damaged ileum villi from pigs. Arginine is the only substrate for NO production in the body [8], and there is no precise method to estimate the dynamics of NO [9]. It has been suggested that the global arginine bioavailability ratio (GABR) can be used as a reliable estimator of arginine homeostasis and the NO synthesis capacity in the body [10,11]. GABR is expressed using the ratio of arginine/(ornithine + citrulline) in the blood [10,12], which is a better indicator of the dynamic changes in arginine and NO in the body. NCG also enhances the antioxidant capacity during the weaning period [13]. In this trial, we aimed to further investigate the effects of dietary arginine or NCG on jejunum development, GABR, and stress-related indices in young rabbits and to evaluate the effects of arginine or NCG on weaning stress and whether arginine meets the organism’s needs. In addition, the possibility of NCG replacing arginine in rabbit production was further analyzed.

## 2. Materials and Methods

### 2.1. Animals

The rabbits in this trial were from the Liuhe rabbits breeding farm at the Jiangsu Academy of Agricultural Sciences. Forty-five litters of newborn rabbits (with does) with similar body weights (58.4 ± 1.55) and sizes (an average of 7 newborn rabbits) were housed in individual cages (66 × 44 × 52 cm) and randomly divided into five groups (n = 9 per group). Each group had 3 replicates (3 does per replicate). They were fed the basal diet (con group), basal diet + 0.3% arginine (0.3% Arg group), basal diet + 0.6% arginine (0.6% Arg group), basal diet + 0.03% NCG (0.03% NCG group), and basal diet + 0.06% NCG (0.06% NCG). The trial lasted 35 days.

### 2.2. Diets and Feeding Procedures

The diet comprised 62% concentrate and 38% grass meal. The ingredients and chemical composition of the basal diet are listed in Table 1. The basal diet was formulated according to the recommended nutrition requirements for rabbits [14]. Arginine and NCG were added to the premix using finely ground maize as a carrier, and the premix was combined with the concentrate. NCG was purchased from the Animore Sci. & Tech Co., Ltd., Beijing, China. Arginine was purchased from Wuxi Jiuxin Biotechnology Co., Ltd., Wuxi, China.

The rabbit cages were cleaned and sterilized before the trial was conducted. Animals were fed twice daily (08:30 and 16:00 h) in two equal portions and given free access to tap water throughout the experimental period. The pellets were 1~2 cm long. Weight and feed intake were recorded once a week.

### 2.3. Sample Collection and Indicator Measurement

On the 36th day, according to the average weight, eight young rabbits with similar body weights (681.4 ± 8.82) were selected from each group. First, their blood samples were collected, and the serum was separated and stored at −20 °C to evaluate the levels of NO, iNOS, cortisol, arginine, ornithine (Orn), citrulline (Cit), following the methods specified in the assay kits from Nanjing Jiancheng Bioengineering Institute, Nanjing, China. Then, they were sacrificed. Carbon dioxide was used for euthanasia of the rabbits. The jejunum tissue was taken; one part was fixed with 4% paraformaldehyde for morphology detection, one part was preserved at −20 °C and used for NO, iNOS, and HSP70 evaluation, and one part was preserved at −196 °C and used for the mRNA expression of iNOS and HSP70.

**Figure 1:**
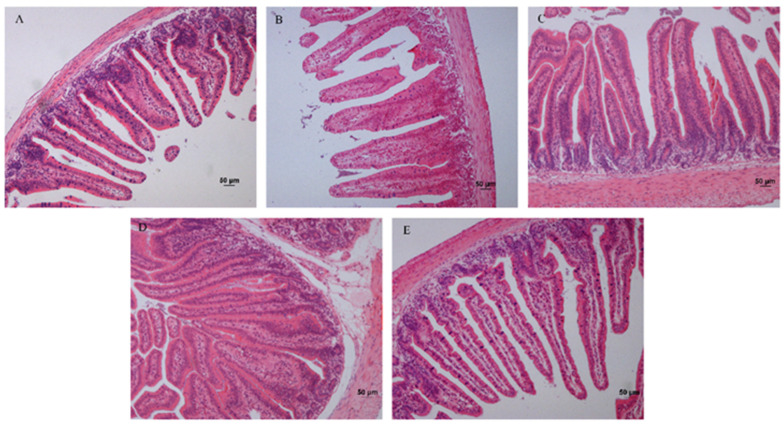
The pathological section of jejunum of 36 d rabbits (original magnification 200×). (**A**) Con group; (**B**) 0.3% Arg group; (**C**) 0.6% Arg group; (**D**) 0.03% NCG group; (**E**) 0.06% NCG group. Bar = 50 μm.

The fixed jejunum tissues were cut into tissue paraffin sections and stained with HE. The sections were observed using an inverted microscope (camera attached) in a 200-fold field view. The Figure 1 showed the morphological structure of the jejunum in each group. Ten field views were randomly collected for each section. Q-capture Pro 6.0 software was used to obtain photographs, and an image-pro plus 6.0 image analysis system was used to measure the height of the intestine villi and the depth of crypts. The selected villi showed an intact morphology and a clear visual field. The length of the villi is the vertical height from the top of the villi to the junction of the villi and the lamina propria. The depth of the crypt is the distance from the indentation of the villous root epithelium to the lamina propria, and 50 measurements are required for each sample.

### 2.4. RT-PCR to Detect the Relative Expression of the mRNA of Jejunal iNOS and HSP70 Genes

The total RNA from the the jejunum tissue was isolated using an RNA extraction kit (Tiangen Biotech (BEIJING) Co., Ltd., Beijing, China). Within the PCR instrument, reverse transcription was performed with a 20 μL system using the Reverse Transcription Kit (Tiangen Biotech (BEIJING) Co., Ltd.). mRNA expression was detected using real-time fluorescence quantitative PCR. The mRNA sequences of β-actin, iNOS, and HSP70 were searched for in GenBank, and the primers were designed using Primer Premier 5.0 software and synthesized by Invitrogen (Shanghai, China) (Table 2).

The RT-PCR system presented was operated under the conditions of a 4 °C ice bath, and the Ct value was detected using the ABI 7500 Real-Time PCR instrument, Thermo Fisher Scientific, Singapore. The specific conditions used are as follows: 95 °C/30 s, (95 °C/5 s and 60 °C/20 s) × 35 cycles, and 72 °C, 34 s. At the end of the reaction, the Ct values of β-actin, iNOS, and HSP70 were obtained. The CT value represents the number of cycles required for the fluorescence signal to reach the threshold for each reaction [ΔCT = CT (target gene) − CT (β-actin)]. The relative expression level of the target gene and β-actin was calculated as 2 ^(−ΔΔCT)^.

### 2.5. Data Analysis

Data were collated using Excel 2016. Data conformed to the normal distribution according to the Shapiro–Wilk test and were statistically analyzed as a completely randomized block using one-way ANOVA in the SPSS 17.0 program. The rabbit cages served as the experimental unit for data. Differences among means were tested using Duncan’s multiple-range tests. Effects were considered significant at *p* < 0.05. Data are expressed as mean ± standard error.

## 3. Results

### 3.1. Jejunum Development in 36-Day-Old Rabbits

Supplementation of arginine or NCG in the diet significantly affected the development of jejunum structure in young rabbits (Table 3). At 36 days, the villus height was higher in the treatment groups (*p* < 0.05) and was significantly higher in the 0.6% Arg group (*p* < 0.01) than in the con group. The crypt depth in the 0.6% Arg and 0.06% NCG groups was lower than that in the control, 0.3% Arg, and 0.03% NCG groups. V/C was higher in the treatment groups than in the con group (*p* < 0.05) and was significantly higher in the 0.6% Arg and 0.06% NCG groups (*p* < 0.01).

### 3.2. Serum NO Concentration and iNOS Activity in 36-Day-Old Young Rabbits

The data on serum NO concentration and iNOS activity are presented in Table 4. At 36 days, the serum NO concentration and iNOS activity were higher in the treatment groups (*p* < 0.05) than in the con group; however, there was no significant difference among the treatment groups (*p* > 0.05).

### 3.3. Jejunum NO Concentration, iNOS Activity, and Gene mRNA Expression in 36-Day-Old Rabbits

Data on the NO concentration and iNOS activity in the jejunum are presented in Table 5. The iNOS activity in the 0.6% Arg group was higher than that in the con group (*p* < 0.05). The NO concentration was high in the 0.6% Arg and 0.06% NCG groups; however, there was no significant difference (*p* > 0.05).

**Figure 2:**
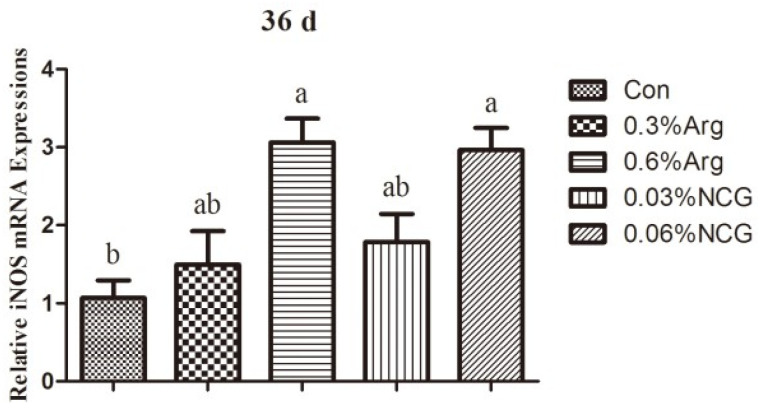
Effects of dietary arginine or N-carbamylglutamate (NCG) supplementation on the mRNA expressions of iNOS in the jejunum of 36-day-old young rabbits. Note: Columns with different letters differ significantly (*p* < 0.05). Data are presented as mean ± standard error (SE) (n = 8). This is the same for Figure 3 and Figure 4.

Figure 2 shows the *mRNA* expression of iNOS in the jejunum of young rabbits. The *mRNA* expression of iNOS was higher in the 0.6% Arg and 0.06% NCG groups than in the control group (*p* < 0.05).

### 3.4. Serum Cortisol in 36-Day-Old Rabbits

**Figure 3:**
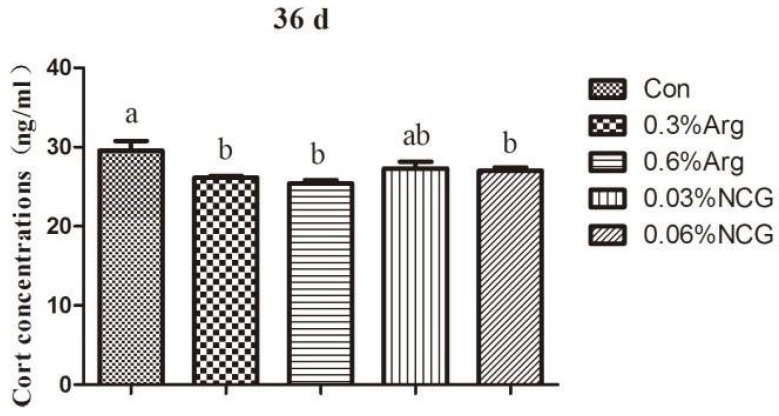
Cortisol concentration in the serum of 36-day-old young rabbits.

Figure 3 reveals the cortisol levels in the serum of 36-day-old rabbits. Compared with the control group, the cortisol concentration was significantly reduced in the 0.3% Arg, 0.6% Arg, and 0.06% NCG groups (*p* < 0.05). 

### 3.5. Concentration and mRNA Expression of HSP70 in the Jejunum of 36-Day-Old Rabbits

**Figure 4:**
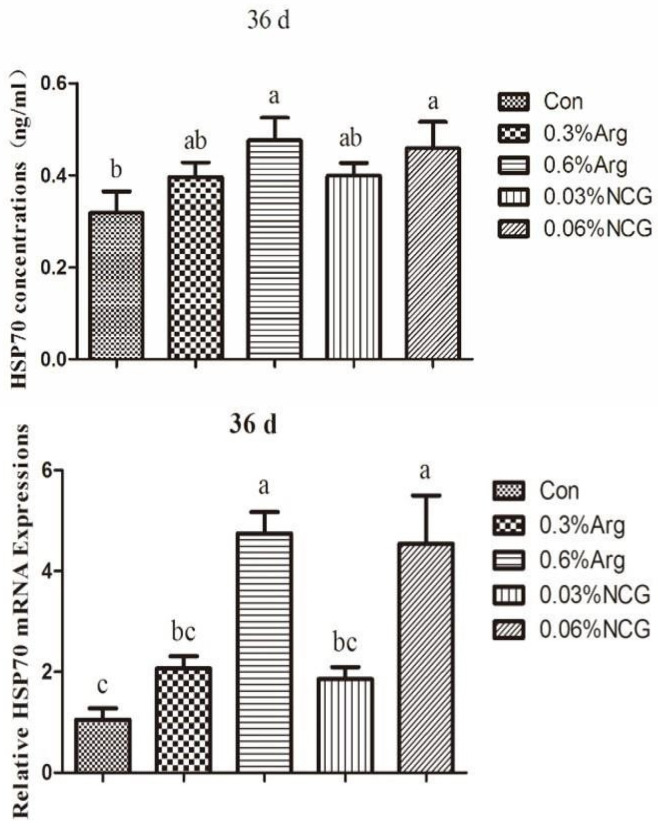
Concentration and mRNA expression of HSP70 in the jejunum of 36-day-old young rabbits.

HSP70 data for the jejunum are shown in Figure 4. The concentration of HSP70 was increased in the treatment groups. The concentration was higher in the 0.6% Arg and 0.06% NCG groups than in the control group (*p* < 0.05). The same results were obtained for the mRNA expression of HSP70. Similarly, the mRNA expression of HSP70 was higher in the 0.6% Arg and 0.06% NCG groups than in the control group (*p* < 0.05).

### 3.6. GABR of Arginine in 36-Day-Old Rabbits

At 36 days old, the level of serum ornithine in the 0.06% NCG group was higher than that in the 0.3% Arg and 0.6% groups (*p* < 0.05), and there was no significant difference in the serum arginine and citrulline levels among the groups (*p* > 0.05) (Table 6). The GABR value was <0.8 and was increased in the treatment groups. It was 16.92% higher in the 0.6% Arg group than in the control group.

## 4. Discussion

When young animals are weaned, the intestine is easily damaged, causing diarrhea and death. This causes serious economic losses in animal husbandry. In rabbit production, weaning stress is more prevalent in young rabbits, and the mortality rate is higher. Therefore, alleviating weaning stress is crucial in nutritional studies. Previous studies have shown that arginine supplementation is beneficial in protecting the normal morphological structure of the intestinal mucosa [15,16], promoting intestinal cell proliferation, differentiation, and maturation [15], and inhibiting intestinal apoptosis [16]. This study revealed that dietary supplementation with 0.6% Arg or 0.06% NCG could significantly increase the height of jejunal villi, decrease the depth of crypts, improve the ratio of villus height to crypt depth, and improve the development of jejunum in young rabbits at 36 days old. Sun [17] reported that dietary supplementation with Arg and NCG could improve the villus height of jejunum and ileum in 65–85-day-old Japanese white rabbits but had different effects on crypt depth. Under stress, arginine, an essential nutritional amino acid for intestinal health, can significantly improve intestinal morphogenesis [18], small intestinal villus height in piglets [19,20], and reduce intestinal damage, inflammation, and oxidative stress [21,22]. Thus, the arginine requirement of weaned piglets is higher than the amount recommended by the NRC [23]. Xiao et al. [24] found that dietary supplementation with arginine, NCG and glutamine (Gln) could maintain the jejunal morphology in mice under oxidative stress. NCG is a potent precursor of arginine and has been clinically used to treat various diseases [25,26]. Dietary supplementation with NCG can promote arginine synthesis in intestinal epithelial cells [27], increase villus height and crypt depth, and improve intestinal atrophy [4]. In addition, NCG can increase the number of intestinal cuprocytes, promote the secretion of mucin, trefoil peptide, and other active molecules, and effectively protect the intestinal mucosal barrier [28,29,30]. Hence, dietary supplementation with arginine or NCG alleviates weaning stress and promotes intestinal development in young animals.

The effect of arginine may be related to the Arg-NO pathway in relieving intestinal stress. In this study, arginine or NCG supplementation increased NO levels and iNOS viability in serum and jejunum tissues and up-regulated the relative mRNA expression of the *iNOS* gene. In mammalian cells, arginine is catalyzed by nitric oxide synthase (NOS) to produce citrulline and NO. NO is an endothelial diastolic factor that can facilitate the maintenance of blood perfusion in intestinal tissues. NO can promote the development of the small intestine and attenuate inflammation or stress damage in tissues. NO reportedly protects the intestinal mucosa from endotoxin damage and maintains the integrity of the vascular bed [31]. Similarly, NO can reportedly induce the production of heat shock proteins in the body [32]. Heat shock proteins are crucial in regulating cell stress, metabolism, proliferation, and death. Adding arginine or NCG to the diet of young animals can promote the production of NO and protect the body and intestinal tract.

When animals are stressed, the hypothalamic–pituitary–adrenal axis is activated, and glucocorticoids are released. It has been shown that weaning stress can increase cortisol levels in piglets [33]. In this study, we detected the serum corticosterone level in rabbits at the time of weaning. At 36 days old, the serum corticosterone level in each experimental group was significantly lower than that in the control group. This suggested that the supplementation of arginine or NCG during the lactation period could reduce corticosterone levels in weaned rabbits and, to a certain extent, improve the body’s immune tolerance of stress. The possible reason is that arginine or NCG supplementation promotes NOS production in the organism and yields more NO, which can inhibit the release of corticotropin-releasing hormone from the hypothalamus through paracrine secretion, reduce corticosterone levels in rabbits, and alleviate stress [34,35]. Similar results were obtained in a study of weaned piglets, which revealed that arginine and NCG could reduce serum cortisol levels [36,37]. This indicates that adding arginine or NCG can enhance the body’s tolerance of weaning stress in young animals. HSP70, a protein with multiple biological functions that can be expressed in gastrointestinal cells, protects the intestinal mucosa by restoring damaged proteins or preventing protein aggregation and denaturation [38,39]. Arginine can induce the body to produce heat shock proteins and enhance the antioxidant capacity of endothelial cells, adipose tissue, and skeletal muscle [40,41,42,43]. In this study, the levels of HSP70 and mRNA expression in the jejunum tissues of weaned rabbits (36 days old) were significantly higher in the 0.6% arginine and 0.06% NCG groups than in the control group. It reveals that arginine or NCG can repair intestinal mucosal injury by increasing HSP70 protein expression in vivo, alleviating intestinal stress and improving intestinal tolerance. Similar results were obtained in weaned piglets [4], which further proves that arginine or NCG can improve or repair intestinal mucosal injury through HSP70 expression. As a signal mediator and vasodilator, NO may be involved in regulating HSP70 expression, and its mechanism needs to be confirmed by further studies.

To better evaluate the arginine utilization status in young animals, the GABR was evaluated in this study. The GABR was calculated by dividing arginine by the sum of ornithine and citrulline. The supplementation of arginine or NCG in the diet could increase the GABR; however, the GABR during weaning was <0.8. In the control group, it was even lower at < 0.7. The GABR has been studied more in medical diseases. Tang et al. [10] reported that the GABR of patients with obstructive coronary artery disease (CAD) was 1.06, and the GABR of patients with non-obstructive CAD was 1.27. Sourij et al. [12] reported that the GABR and the arginine-to-ornithine ratio are associated with markers of endothelial dysfunction and an increased risk of cardiovascular mortality. Ali-Sisto et al. [44] found that the GABR was decreased in patients with major depressive disorder; this could impair NO production and increase oxidative stress in the central nervous system. Therefore, in this trial, it was inferred from the GABR that the body would possibly experience more inflammatory stresses at 36 days old. Arginine cannot be synthesized at the litter stage, and the more arginine is required for young animals, the more the addition of arginine may alleviate the onset of intestinal inflammation. Bersani et al. [45] reported that the GABR is a marker of NO synthesis capacity and inflammation, and in Post-Traumatic Stress Disorder (PTSD), the GABR was decreased and correlated with symptom severity. Krzystek-Korpacka et al. [46] found that chronic wounds during cardiometabolic diseases are associated with reduced NO and arginine availability, and wound character seems to affect NO bioavailability and wound etiology–arginine bioavailability. Arginine concentration and its availability are more significantly reduced at the local level than at the systemic level. Miyazaki et al. [47] found that arginine bioavailability was reduced in patients with CAD in an outpatient setting, and the metabolic profile of arginine, particularly Arg/Cit and GABR, has independent predictive value for the presence of CAD over traditional risk factors. This shows that the GABR is crucial for predicting diseases and also reflects the effect of arginine or NO on the body.

In this study, the effects of adding 0.6% arginine or 0.06% NCG were similar; however, from the GABR viewpoint, adding 0.6% arginine or 0.06% NCG could not meet the needs of weaned animals. However, in livestock production, the price of NCG is similar to that of arginine, but the added amount is only 1/20–1/10. Thus, the cost of arginine is significantly higher than that of NCG. Therefore, NCG can be added to the diets of young animals as a substitute for arginine, which can effectively reduce production costs.

## 5. Conclusions

In this study, we found that the levels of arginine in the bodies of young rabbits was far from meeting their needs based on the GABR. Dietary supplementation with arginine or NCG could alleviate this situation. Firstly, adding arginine or NCG significantly increased the iNOS level, up-regulated the relative mRNA expression of the iNOS gene in jejunum tissues, promoted NO synthesis, and further improved jejunum morphogenesis. Secondly, arginine and NCG significantly reduced serum corticosterone levels, increased HSP70 content, and up-regulated the relative mRNA expression of the HSP70 gene in jejunum tissue. This indicates that arginine and NCG can improve the body’s tolerance of weaning stress and improve or repair intestine mucosal damage. Analyses regarding arginine bioavailability and jejunum morphogenesis revealed that adding 0.6% arginine and 0.06% NCG at the lactation stage may alleviate intestinal inflammation and arginine deficiency of young rabbits. Adding NCG yields results that are similar to those for arginine; hence, NCG can be used instead of arginine during rabbit production. Moreover, the cost of adding NCG is lower than that of arginine.

## Figures and Tables

**Table 1:** Composition and nutrient levels of the diet.

Ingredients	Rate (%)	Nutrient Levels	
Corn	22.0	Digestible energy (DE; MJ/kg) ^2^	10.17
Wheat bran	20.9	Crude protein (CP; %)	16.19
Soybean meal	10.8	Crude fiber (CF; %)	11.57
Rapeseed meal	3.0	Crude fat (EE; %)	3.65
Salt	0.3	Calcium (Ca; %)	1.54
Chrysanthemum powder	38	Effective phosphorus (AP; %)	0.40
Premix ^1^	5.0	Lys (%)	0.96
		Met + Cys (%)	0.47
Total	100	Arg (%)	0.82

^1^ Per kilogram of premix contained FeSO_4_·H_2_O (5320 mg), CuSO_4_·5H_2_O (1080 mg), MnSO_4_·H_2_O (560 mg), ZnSO_4_·H_2_O (3652 mg), CoCl_2_·6H_2_O (1000 mg), VA (180,000 IU), VD (18,000 IU), VE (900,000 IU), CaHPO_4_ (250 g), Lys (60 g), and Met (40 g). ^2^ DE was estimated through total energy, while the others were measured values.

**Table 2:** Sequences of primers for real-time fluorescence quantitative PCR (qRT-PCR).

Target Genes	Accession No.	Primer Sequence	Product Length
β-actin	NW_003159504.1	F: 5′-GGCTACAGCTTCACCACCAC-3′	496 bp
		R: 5′-ACTCCTGCTTGCTGATCCAC-3′	
iNOS	NC_013687.1	F: 5′- GCTACACTTCCAACGCAACA-3′	161 bp
		R: 5′- GCGGCTGGACTTCTCACTAT-3′	
HSP70	NC_013671.1	F:5′-GAGTGAGGAGAGGCGTCAGT-3′	199 bp
		R:5′-GTTCTCACACAGGTCGGACA-3′	

**Table 3:** Effects of dietary arginine or N-carbamylglutamate (NCG) supplementation on jejunum development in 36-day-old young rabbits.

Item	Con Group	0.3% Arg Group	0.6% Arg Group	0.03% NCG Group	0.06% NCG Group
Villus height (μm)	351.67 ± 12.25 ^c^	419.27 ± 11.22 ^b^	461.33 ± 9.16 ^a^	418.17 ± 13.31 ^b^	433.14 ± 10.58 ^ab^
Crypt depth (μm)	80.81 ± 3.61 ^a^	80.70 ± 2.21 ^a^	68.80 ± 3.03 ^b^	78.72 ± 2.10 ^a^	70.53 ± 2.96 ^b^
Villus height/Crypt depth (V/C)	4.42 ± 0.0.18 ^c^	5.26 ± 0.20 ^b^	6.95 ± 0.43 ^a^	5.36 ± 0.21 ^b^	6.35 ± 0.34 ^a^

Note: In the same row, values with no letter or the same letter superscripts indicate no significant difference (*p* > 0.05), while those with different small letter superscripts indicate significant differences (*p* < 0.05). This is the same for the table below.

**Table 4:** Effects of dietary arginine or NCG supplementation on serum NO concentration and iNOS activity in 36-day-old young rabbits.

Item	Con Group	0.3% Arg Group	0.6% Arg Group	0.03% NCG Group	0.06% NCG Group
NO (μ mol/L)	19.59 ± 2.31 ^b^	32.28 ± 1.01 ^a^	30.27 ± 1.25 ^a^	30.03 ± 1.02 ^a^	32.29 ± 4.76 ^a^
iNOS (U/mL)	9.85 ± 0.63 ^b^	13.25 ± 1.27 ^a^	13.65 ± 1.81 ^a^	13.65 ± 1.91 ^a^	15.94 ± 0.21 ^a^

**Table 5:** Effects of dietary arginine or NCG supplementation on NO concentration and iNOS activity in the jejunum of 36-day-old young rabbits.

Item	Con Group	0.3% Arg Group	0.6% Arg Group	0.03% NCG Group	0.06% NCG Group
NO (μ mol/gprot)	0.32 ± 0.04	0.32 ± 0.09	0.41 ± 0.02	0.37 ± 0.03	0.42 ± 0.03
iNOS (U/mgprot)	0.80 ± 0.05 ^b^	1.06 ± 0.23 ^ab^	1.58 ± 0.13 ^a^	1.33 ± 0.19 ab	1.50 ± 0.18 ^a^

**Table 6:** Effects of dietary arginine or NCG supplementation on global arginine bioavailability ratio for 36 days.

Item	Con Group	0.3% Arg Group	0.6% Arg Group	0.03% NCG Group	0.06% NCG Group
Cit (nmol/mL)	27.18 ± 2.47	23.63 ± 2.19	25.95 ± 3.25	25.45 ± 1.38	23.99 ± 1.32
Orn (nmol/mL)	261.13 ± 13.93 ^ab^	230.48 ± 10.40 ^b^	226.78 ± 12.14 ^b^	251.43 ± 12.96 ^ab^	272.19 ± 6.88 ^a^
Arg (nmol/mL)	188.28 ± 11.45	179.04 ± 16.80	191.52 ± 7.04	199.89 ± 7.42	205.15 ± 3.11
BABR	0.65 ± 0.01	0.71 ± 0.09	0.76 ± 0.03	0.73 ± 0.03	0.69 ± 0.02

## Data Availability

Data are contained within the article, and the research data supporting this study will be shared upon a reasonable request made to the corresponding author. The data are not publicly available due to privacy.
